# Intraneural perineurioma: a retrospective study of 19 patients

**DOI:** 10.11604/pamj.2018.30.275.16072

**Published:** 2018-08-14

**Authors:** Jaber Alkhaili, Adeline Cambon-Binder, Zoubir Belkheyar

**Affiliations:** 1 Department of Orthopaedic Surgery, Bichat Claude Bernard Hospital, Paris, France; 2 Hospital Saint Antoine, Paris, France; 3 Clinic of Mont Louis, Paris, France

**Keywords:** Intraneural perineurioma, hypertrophic neuropathy, pseudo-onion bulbs

## Abstract

Intraneural perineurioma is a benign neoplasm of peripheral nerve sheath with perineurial cell origin that typically affects teenagers and young adults and tends to result in a motor-predominant neuropathy. The aim of our study is to present the epidemiology, clinical presentation, way of diagnosis and management plan in a consecutive patient series. Ninteen patients diagnosed as having intraneural perineurioma were retrospectively chart reviewed. Diagnosis was done by MRI and/or biopsy with morphological, immunohistochemical staining study confirmation. Patients assessments included gender, age, symptoms, tumor localization, radiological aspect, management and pathological confirmation.Sex ratio was 10 males to 9 females with mean age of 31.2 (15-64). All the patients presented with motor deficit, ten with sensory deficit. Upper limb was involved in 11 cases (among them 4 lesions of brachial plexus), the lower limb in 8 cases. On magnetic resonance imaging, 16 patients showed a nerve enlargement whereas 5 presented with atypical fusiform tumor. Eighteen patients were operated for excision biopsy and/or palliative treatment for their motor deficit. Anatomopathological analysis confirmed the diagnosis in seventeen cases with a morphological pseudo-onion bulb shape and/or specific immunohistochemical assay. One patient had only palliative treatment without excision biopsy. Our data confirmed the equal penetration of intraneural perineurioma to both sex and affected limb. Because of the benignity of the tumor, the surgical treatment focused on optimizing the functional outcome. A prospective study with long term follow-up is required for this under-diagnosed tumor.

## Introduction

Perineuriomas are divided mainly into two forms: intraneural and extraneural perineuriomas. Intraneural perineurioma was first identified in 1964 [1, 2]. It represents 1 to 5% of neural tumors.and this is probably due to the unfamiliarity of physicians with this condition [3, 4]. It was thought to be a post-traumatic phenomenon; however it is now believed to be neoplastic process [2, 5]. Intraneural perineurioma affects commonly young adults, equally males and females [6]. Patients typically present with equal distribution between upper and lower limbs with gradual onset of a motor-predominant neuropathy which may accompanied with a mild sensory component [7]. The pathological examination of perineurioma has demonstrated a distinctive nerve lesion. The perineurial tumor is composed of concentric layers of perineurial cells [4] These concentric layers of cells resemble the onion-bulb appearance in Schwann cell lesions. However the Schwann cell lesions arise from endoneurial layers and perineurioma arise from perineurial layer which made the term pseudo-onion bulb lesion more suitable and acceptable for the perineuriomas [4, 8] The immunohistochemical staining can help to distinguish between perineurioma and Schwann cell lesion as the first is positive for epithelial membrane antigen (EMA) and negative for (s-100) and the Schwann cell lesions demonstrate the opposite pattern [4]. Some genetic abnormalities in perineurioma have been linked to the long arm of chromosome 22, known to contain the abnormalities in schwannoma and neurofibromatosis [5]. The aim of our study was to present the epidemiology, clinical presentation, way of diagnosis and management plan in a consecutive patient series.

## Methods

This monocenter study was lead retrospectively after appropriate consentment and ethics committee aproval. We included all patients diagnosed of Intraneural perineurioma by MRI and/or biopsy with morphological, immunohistochemical staining study confirmation between 2009-2014. Assessment of the patient charts included gender, age, symptoms, tumor localization, radiological aspect, pathological confirmation and management with the therapeutical strategy .

## Results

Nineteen patients were included in this study. Their demographic and clinical features were summarized (Table 1). The median age on onset of symptoms was 31.2 years (range from 15 to 64 years). There were 10 males and 9 females. All the patients complained from motor dysfunction. Ten patients had also a sensory deficit (52.6%). Upper limb was involved in 11 cases (among them 4 lesions of brachial plexus), the lower limb in 8 cases (5 peroneal nerves and 3 sciatic nerve). There were 9 patients affected on the right side of the body and 10 on the left side. All patients underwent MRI. An aspecific nerve enlargement was seen in 16 cases. Five patients presented with a typical fusiform tumor (hypointense to isointense on T1-weighted images and hyperintense on T2-weighted images). The tumors size ranged from 1 to 10 centimeters. Eighteen patients were operated for excision biopsy and/or palliative treatment for their motor deficit. In 17 patients, pathoanatomy analysis confirmed the diagnosis with a morphological pseudo-onion bulb shape and/or specific immunohistochemical assay of (+) EMA and (-) s 100. 1 patient had only palliative treatment without excision biopsy.

**Table 1:** 19 patients and their demographic and clinical features summarized

	Age (y)	Sex	Affected nerves	Side	Motor Deficit	Sensitive Deficit	Size	MRI	Immunohistochemical staining + pathology
1	63	F	Radial	R	+	-	1.5	+	
2	17	F	Brachial Plexus +Axillary	L	+	+	1.3	+	EMA (+) S100 (-) EGFR (+) , pseudo ognion bulb tumor
3	27	M	Peroneal	L	+	+	1.5	+	EMA (+)
4	22	F	Peroneal	L	+	-	6	+	EMA (+) S100 (-)
5	18	M	Radial	R	+	-	1	+	EMA ( atypic) S100 (-) , pseudo ognion bulb tumor
6	40	F	Peroneal	L	+	-	2	+	EMA ( difficult) , pseudo ognion bulb tumor
7	37	F	Axillary	L	+	-	2	+	EMA (+) , pseudo ognion bulb tumor
8	20	M	Peroneal	L	+	-	4.8	+	EMA (+) , pseudo ognion bulb tumor
9	17	F	Brachial Plexus	L	+	+	-	+	TT paliative
10	45	F	Sciatic	L	+	+	10	+	
11	45	F	Sciatic	R	+	+		+	EMA (+) S100 (+) pseudo ognion bulb tumor
12	31	M	Sciatic	R	+	+		+	
13	15	M	Brachial plexus	R	+	+	4,7.5,2	+	EMA (+) S100 (+) pseudo ognion bulb tumor
14	49	F	Median	L	+	+	10	+	
15	29	M	Radial	R	+	-		+	
16	21	M	Peroneal	L	+	-	4	+	EMA (+) S100 (+) pseudo ognion bulb tumor
17	18	M	Brachial Plexus	R	+	+	6	+	pseudo ognion bulb tumor clinically
18	37	M	Median	R	+	+		+	pseudo ognion bulb tumor clinically
19	23	M	Musculocutaneous	R	+	-	6	+	pseudo ognion bulb tumor

## Discussion

**Figure 1:**
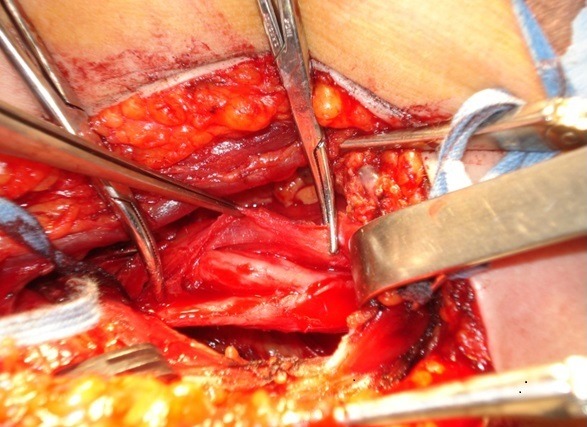
Case of a 17 years old female with tumor of the posterior trunk of the left brachial plexus with fully paralyzed deltoid muscle

**Figure 2:**
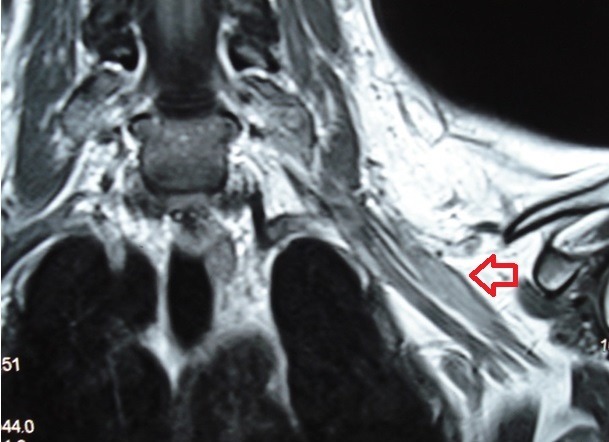
MRI showing typical aspect of hyperintense T2-weighted images on the posterior trunk of the left brachial plexus

**Figure 3:**
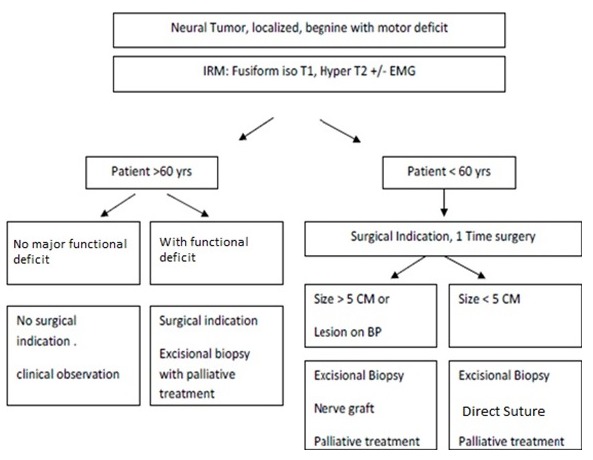
Surgical decision-making tree

Most of the perineurioma cases presented in the literature were single case reports or small case series. Literature review in 2014 presented a total of 120 cases [9]. Mauermann compiled the longest series of 32 cases reported since 1995 [10]. Typically this tumor presented at the age of adolescence or early adulthood [9]. However In our series, the average age of patients was higher than in other series [5, 10, 11 ]. Our data confirmed the equal penetration to both sex and involved limbs [10] despite other studies reporting predominance in the upper limb [5]. It also confirmed motor involvement of the tumor. However, it showed frequent sensory deficit association [9]. In our series, the average age of patients was higher than in other series [5, 10, 11]. The MRI usually is a main tool for preoperative evaluation and for limiting the differential diagnosis in patients with clinical presentation.The fusiform enlargement with preserved fascicular architecture can be seen in lipomatosis with different characterstic in MRI. It can be seen also in schwannoma and neurofibroma with more rounded appearance [4] .In intraneural perineurioma usually the tumor present as hypointense to isointense on T1-weighted images and hyperintense on T2-weighted images [9].Although the main definitive way to confirm the diagnosis is the biopsy, it has been suggested that a typical clinical presentation with appropriate MRI and nerve conduction study can made the diagnosis [12] . In our series it was of little help as most of patients showed non specific enlargement (Figure 1, Figure 2) Intraneural perineurioma management is contraversial, due to its natural history and long term prognosis. It is considered as benign tumor without the potential of malignancy [5].However, it can progress to severe functional disability as some patients with partial nerve involvement can progress to full nerve dysfunction [8]. It has been proven that surgical resection with direct suture for short segment or nerve grafting for the long segment give better outcomes with mixed results in the literatures [10, 13, 14]. Our surgical strategy focused to optimize the functional outcome, as the tumor is benign with special attention to patient's age and the size of the tumor (Figure 3) For the patients below the age of 60 years old with typical clinical presentation and MRI confirmation, one time surgery was proposed. The surgery consist of excisional biopsy and direct suture if the lesion shorter than 5cm. However, if the lesion larger than 5cm and brachial plexus involved a nerve graft was proposed with most of the time palliative treatement for the affected nerve. For the patients above the age of 60 years old, we tend to be more conservative unless there is a major functional deficit in which we propose the surgery with the palliative treatement.

## Conclusion

Diagnosis of intraneural perineurioma requires multidisciplinary experts in peripheral nerves imaging, pathology and surgery. This tumor should be suspected for any slow progression motor deficit with or without sensory components, with focal hyperintense lesion on T2-weighted MRI images. The surgical decision should be taken in relation with patient age and functional deficit.

### What is known about this topic

- Intraneural perineurioma is a benign neoplasm;
- This tumor typically affects teenagers and young adults;
- Usually result in a motor-predominant neuropathy.

### What this study adds

- This study represents one of the large clinical series of intraneural perineurioma in the literature (most of them are case studies);
- It shows high sensory component that can be accompanied in this type of tumors;
- It shows that this tumor does not affect adults only.

## Competing interests

The authors declare no competing interest.
